# Usefulness of 3T MR in surgical planning for deep brain stimulation surgery: a systematic literature review

**DOI:** 10.1007/s10143-025-03561-7

**Published:** 2025-05-13

**Authors:** Quintino Giorgio D’Alessandris, Francesco Pambianco, Renata Martinelli, Manuela D’Ercole, Francesca Annunziata, Carla Piano, Francesco Bove, Maria Filomena Fuggetta, Alessandro Izzo, Nicola Montano

**Affiliations:** 1 Department of Neuroscience, Università Cattolica del Sacro Cuore, Roma, Italy; 2 Department of Neurosurgery, Fondazione Policlinico Universitario A. Gemelli IRCCS, Largo A. Gemelli 8, Roma, 00168 Italy; 3 Department of Neurology, Fondazione Policlinico Universitario A. Gemelli IRCCS, Roma, Italy

**Keywords:** Deep brain stimulation, Magnetic resonance imaging, Direct targeting, 3T

## Abstract

Magnetic resonance imaging (MRI) allows direct visualization and targeting of the subthalamic nucleus (STN) and the globus pallidus interna (GPi) during deep brain stimulation (DBS) surgery for Parkinson’s disease (PD). Compared to standard 1.5T MRI, the availability of MRI machines with higher magnetic field strength, as 3T MRI, could provide surgical advantages for DBS surgery. The aim of the present systematic review was to gather the available evidence on targeting precision and accuracy, and on clinical outcome, of DBS performed using 1.5T vs. 3T MRI. The literature search yielded 289 results. After duplicate removal and title and abstract screening, 15 full-text papers were assessed, ultimately resulting in the inclusion of six studies in the present work. An improved visualization of STN with 3T MRI was described. Two studies analyzed targeting precision, finding no difference between 1.5T and 3T. Targeting accuracy was evaluated using microelectrode recording-based nucleus identification in four studies: three reported an increased accuracy using 3T MRI, and one reported no differences. Clinical outcome was assessed in three papers, and was judged similar between 1.5T and 3T-based DBS. Risk of bias from the included studies was non-negligible. In conclusion, while the use of 3T MRI can foster deep gray nuclei identification during DBS, according to the available evidence the use of 1.5T MRI remains an adequate option. Further research on this topic is needed.

Clinical trial number: Not applicable.

## Introduction

Deep brain stimulation (DBS) surgery is considered the treatment of choice for patients suffering from drug-resistant movement disorders, such as Parkinson’s Disease (PD) [6]. The main targets are the subthalamic nucleus (STN) and the globus pallidus interna (GPi) [10]. Preoperative targeting of these structures has been traditionally performed using standard atlas-based coordinates [9]; in recent years, however, the improvement in diagnostic images accuracy has made possible to directly visualize the STN and the GPi on magnetic resonance imaging (MRI), thus allowing a direct targeting of these structures [9]. Direct visualization of STN was first described by Bejjani et al. in 2000 using a 1.5T MR scanner [2], a technology which is currently available virtually in all neurosurgery departments devoted to DBS. However, reportedly, STN borders can be difficult to identify on 1.5T MRI [15]; the use of higher-field imaging, namely 3T MRI, instead, allows an improved visualization of STN [15]. A similar reasoning applies also to GPi, notwithstanding its larger volume [16]. Though the first reports on 3T use in clinics for brain imaging date back to early ‘00s [7], 3T scanners are still not universally available at all neurosurgical facilities. Further, the advantages of performing presurgical planning on 3T MRI, rather than on 1.5T MRI, is still an open issue in the literature.

In the present work, with the aim of clarifying this issue, we performed a systematic review of studies comparing precision, accuracy and outcomes of DBS using 1.5T vs. 3T MRI as baseline scan for surgical targeting.

## Methods

A search was launched in the PubMed, Scopus and Embase databases to identify papers comparatively addressing the use of 1.5T and 3T MRI for STN or GPi DBS planning and targeting. We designed the PICO question as follows: “For patients suffering from PD and undergoing STN or GPi DBS surgery (P), does targeting based on 3T MRI (I), compared with 1.5T MRI (C), produce improved outcomes in terms of precision, accuracy, clinical effectiveness or complication rate (O)?”

The search string was: ((deep brain stimulation) OR (DBS) OR (Parkinson) OR (Parkinson’s)) AND (3T OR 3-T OR 3-Tesla OR “3 Tesla”) AND (1.5T OR 1.5-T OR 1.5-Tesla OR “1.5 Tesla”), in any possible combination. PRISMA guidelines were followed [11]. Studies comparing STN or GPi targeting during DBS procedures for PD using 1.5T vs. 3T MRI were included, irrespective of the type of targeting (direct, indirect or mixed). We excluded: *(i)* studies focused on targeting of the ventral intermediate nucleus of the thalamus (VIM), not directly visible on 1.5T or 3T MRI; *(ii)* non-comparative studies; *(iii)* studies not reporting original data; *(iv)* studies performed on phantoms or models, not including real patients; *(v)* studies written in languages other than English. No limitations on data publication were adopted. Last search was launched in January 2025. Three Authors (Q.G.D., F.P. and R.M.) independently performed the search; controversies were solved by discussion with a senior Author (N.M.).

The risk of bias was assessed using the ROBINS-I (Risk of Bias in Non-randomized Studies of Interventions) assessment tool (available at https://mcguinlu.shinyapps.io/robvis/).

## Results

**Fig. 1:**
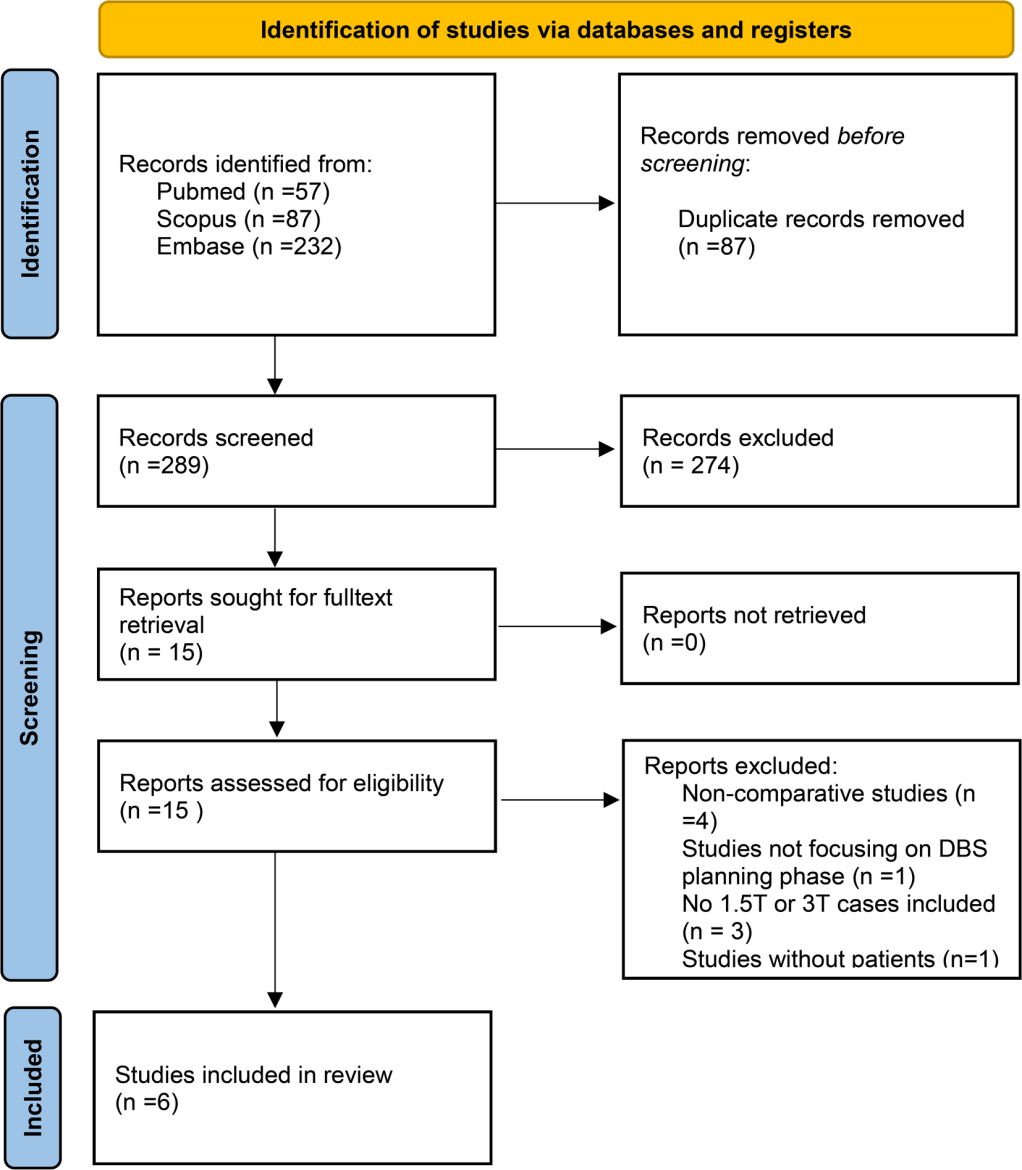
PRISMA flow diagram

After duplicate removal, 289 records were identified for screening. Of these, 274 were found to be not relevant for the present work based on title and abstract. Fifteen full text papers were thus assessed, of which 9 were excluded. The reasons for exclusion were the following: papers described non-comparative studies (*n* = 4), studies not assessing DBS planning (*n* = 1), studies lacking 1.5T or 3T cases (*n* = 3), or phantom-only studies (*n* = 1). In conclusion, six studies were included in the present systematic review (Fig. 1).

Details on the included studies are provided in Table 1.

The first study to address the issue on the use of 1.5T vs. 3T MRI for DBS targeting was published by Winkler et al. in 2006 [21]. They prospectively enrolled 27 patients candidate to frame-based STN DBS performed using a mixed targeting. For STN visualization, 1.5T T1w images were used as reference and were fused to 1.5T (*n* = 13) or 3T (*n* = 14) T2w images. Using 3T T2w MRI, the Authors reported a better visualization of STN with improved targeting. Accordingly, they found an increase in the number of typical STN electric activity during microelectrode recording (MER)– from 6.7 to 7.1 per patient. Of note, they used a multitrack MER technique in which they systematically recorded all 5 MER tracks in each side. However, other parameters, like image fusion accuracy and, more importantly, levodopa equivalent dose reduction (LEDD) were not significantly different between the two groups.

**Table 1:** Systematic review of studies comparing DBS targeting using 1.5T vs. 3T MRI

Author, Year	Type of Study	*N* patients	Frame	Target	Type of Targeting	Results
Winkler, 2006 [21]	Pros	13 (1.5T group) 14 (3T group)	yes (Zamorano-Dujovny)	STN	mixed	No significant differences image fusion accuracy and in LEDD reduction. Increase of typical STN MER signal using 3T.
Acar, 2007[1]	Retros	20 undergoing both 1.5T and 3T imaging	yes (Leksell)	STN	indirect (if 1.5T) direct (if 3T )	No significant differences (< 1 mm) in STN location coordinates between indirect 1.5T-based targeting and direct 3T-based targeting
Toda, 2009 [][17]	Pros	13 (1.5T group) 13 (3T group)	yes (Leksell)	STN	Different types (indirect, RN-based, and composite)	Longer lead path length in STN and increased use of central trace in 3T-based vs. 1.5T-based targeting. Similar clinical outcome.
Cheng, 2014[4]	Retros	16 (1.5T group) 23 (3T group)	yes (Leksell)	STN	mixed (indirect MCP-based, then direct)	STN visualization improved using 3T vs. 1.5T images. Use of 3T resulted in less trajectories and reduced surgical time compared to 1.5T. Similar clinical outcome.
Houshmand, 2014[8]	Retros	10 undergoing both 1.5T and 3T imaging	yes (Leksell)	STN	indirect (RN-based)	No significant differences in STN location coordinates between 1.5T-based and 3T-based indirect targeting.
Southwell, 2016 [][14]	Retros	15 (1.5T group) 12 (3T group)	no	STN and GPi	direct (iMRI ClearPoint)	No significant differences in radial error, number of traces and surgical time between 1.5T-based and 3T-based targeting. Better target visualization with 3T images.

GPi, globus pallidus interna; LEDD, levodopa equivalent daily dose; MCP, midcommissural point; MER, microelectrode recording; Pros, prospective; Retros, retrospective; RN, red nucleus; STN, subthalamic nucleus

In a subsequent work by Acar et al. (2007) [1], 20 patients undergoing STN-DBS for PD were retrospectively analyzed focusing on the precision of STN targeting, using either 1.5T MR-based indirect targeting or 3T MR-based direct targeting. In both groups, the reference exam was 1.5T T1w image, but in the second group it was fused with a 3T T2w MRI. Notably, the distance between atlas-based and directly identified *x* and *y* STN coordinates was < 1 mm in the vast majority of cases, while for the z coordinate such distance was < 1 mm in 55% cases. The Authors conclude that indirect atlas-based STN targeting remains a viable option, provided the frame is aligned to the AC-PC plane. No clinical data are provided in this study.

Toda et al. (2009) [17] reported on 26 prospective patients undergoing bilateral, frame-based (Leksell) STN-DBS, in whom targeting was performed on pre-operative 1.5 T (*n* = 13) or 3T (*n* = 13) MRI. In these patients, the Authors used a novel and complex direct targeting method, called “modified composite targeting”; it was based on the identification of several structures, namely the post-mammillary commissure, the mammillothalamic tract, the red nucleus (RN), and the anterior part of the STN. They reported that the use of 3T MR resulted in an increased STN length on the central track, as assessed using MER (4.9 mm vs. 3.1 mm using 1.5T) and in an increased use of the central track for final electrode placement (81% using 3T vs. 31% using 1.5T). From a clinical viewpoint, reduction in off-med and on-med Unified Parkinson’s Disease Rating Scale (UPDRS) III score and in LEDD was slightly superior using 3T vs. 1.5T MR, with this difference being not statistically significant. Moreover, no differences in complications rate were observed.

Similar results were obtained by Cheng et al. (2014) [4], who retrospectively reviewed 39 PD patients operated on for STN-DBS. They performed frame-based DBS using mixed targeting, in which indirect coordinates where adjusted based on direct STN visualization, using as pre-op MRI a T2w image acquired on 1.5T (*n* = 16) or 3T (*n* = 23) scanner. Firstly, they semiquantitatively scored the clearness of STN contour at MRI on a 3-point scale (0, non-visible; 1, visible with blurred margin; 2, clearly visible). As expected, all 3T MR images were assigned a score 2, while the mean score in the 1.5T group was 1.19 (*p* < 0.001). Procedures were performed using multi-step MER recording: patients in the 3T group had a significantly reduced mean number of MER tracks (1.2 vs. 1.5 in 1.5T group, *p* = 0.0049) and consequently a shorter surgical time (233 min vs. 309 min in 1.5 T group, *p* = 0.003). Complication rate was not statistically different. Also in this case, however, improvement in UPDRS III motor score after DBS surgery was similar between 3T and 1.5T MR groups.

Houshmand et al. (2014) [8] performed an elegant study focused on the comparison of two indirect targeting methods, midcommissural point-based and RN-based, in STN DBS for PD, demonstrating the superiority of the latter over the former. In a subgroup of 10 patients, they compared the STN coordinates calculated on 1.5T vs. 3T MRI, finding no significant differences. It should be noticed, however, that the indirect targeting method they describe rely on the identification of RN, which is usually well visible also in 1.5T images, and not of STN.

Finally, Southwell et al. (2017) [14] analyzed a cohort of 27 patients operated on for STN DBS or for GPi DBS using a frameless technique (ClearPoint system) in which direct targeting was performed in the operating room using intraoperative MRI. In this cohort, 15 patients underwent 1.5T MRI and 12 patients underwent 3T MRI. Though 3T MRI allowed superior target visualization compared to 1.5T MRI, no significant differences were found regarding targeting coordinates (radial error), number of trajectories and duration of surgery. Again, no clinical data are provided.

**Fig. 2:**
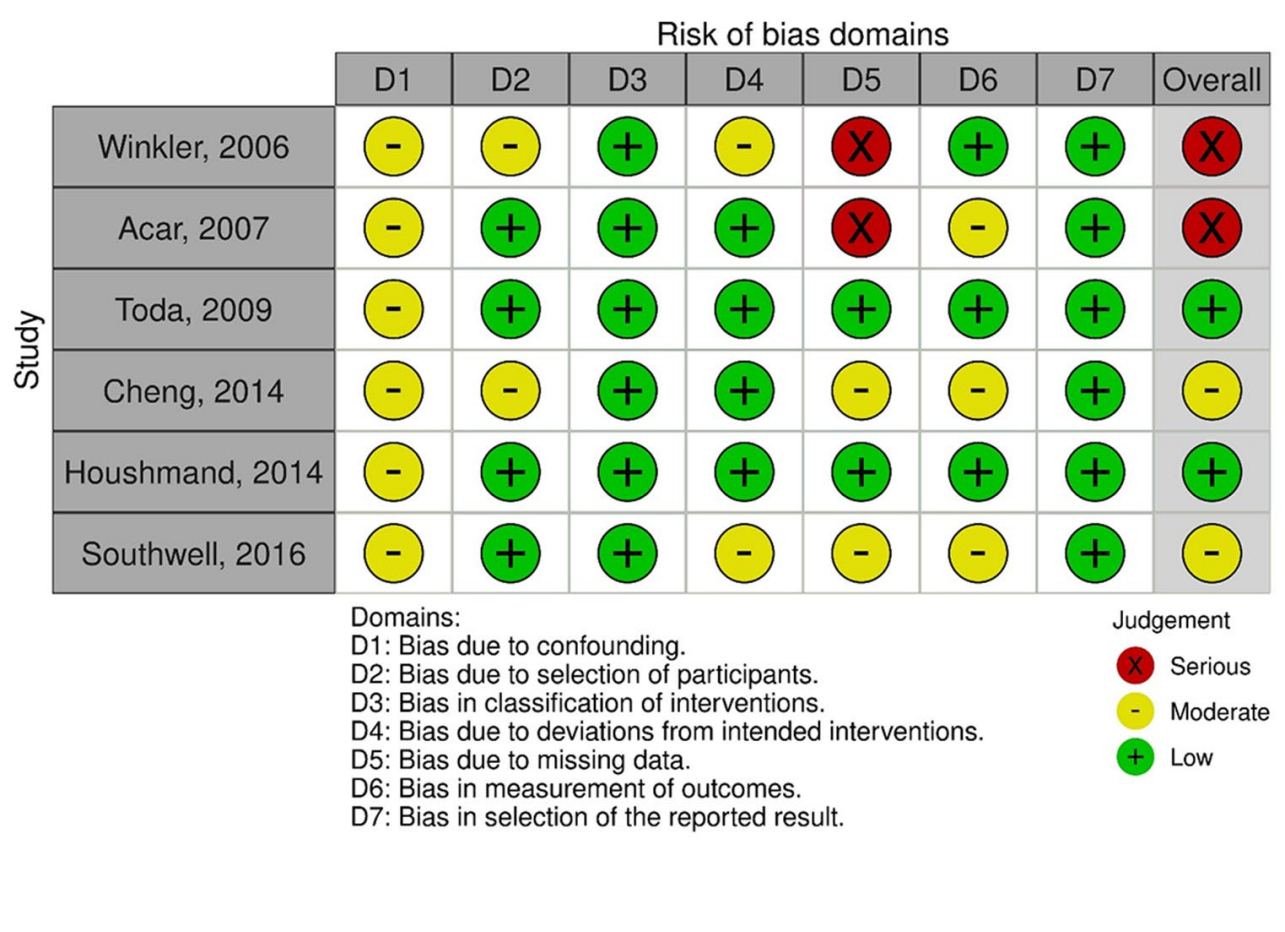
Risk of bias assessment

Risk of bias, assessed using the ROBINS-I tool, is presented in Fig. 2. It was judged serious in the two older studies, moderate in two studies and low in other 2 studies.

## Discussion

While DBS surgery has benefited from the huge technological improvement of latest years, the actual impact of innovation is difficult to quantify. In this regard, we performed the present systematic review to assess the usefulness of high-field 3T MRI in routine DBS procedures.

### Evidence from the systematic review

Our systematic review found six studies only comparing DBS targeting using 1.5T vs. 3T MRI. Unfortunately, the studies were highly heterogeneous in terms of DBS technique and, more importantly, of measured outcomes, thus a formal metanalysis could not be performed.

**Table 2:** Summary of findings from the literature review

Parameter	Result (3T vs. 1.5T MR)	Reference
Sharpness of target borders	Improved	Cheng, 2014 [4]; Southwell, 2017 [14]
Image fusion accuracy	No difference	Winkler, 2006 [21]
Targeting precision	No difference	Acar, 2007 [1]; Housmand, 2014 [8]
Targeting accuracy	Increased	Winkler, 2006 [21]; Toda, 2009 [17]; Cheng, 2014 [4]
	No difference	Southwell, 2016 [14]
Surgical time	Reduced	Cheng, 2014 [4]
	No difference	Southwell, 2016 [14]
Clinical outcome	No difference	Winkler, 2006 [21]; Toda, 2009 [17]; Cheng, 2014 [4].

Nonetheless, we were able to identify some aspects such as the sharpness of target borders, the image fusion accuracy, the targeting precision, the targeting accuracy, the surgical time and the clinical outcome (see Table 2) that could be separately discussed in order to gather some evidence on this topic.

**Table 3:** Main studies addressing the use of 7T MRI during DBS surgery

Author, Year	Type of Study	*N* patients	Frame	Target	Type of Targeting	Results
Damman, 2011[5]	Pros	phantom study	No	centre of phantom	NA	Hardware-related distortions with 7 T MRI lower than expected.
Van Laar, 2017[][18]	Pros	3 (1.5T, 3T, 7T)	NA	STN, RN	Direct	7T MRI not superior to 3T MRI in the surgical accuracy of DBS targeting.
Verhagen, 2017[19]	Retros	8 (1.5T group) 5 (3T group) 6 (7T group)	Yes (Leksell)	STN	Mixed	Discrepancies between MER-derived and MRI-derived STN borders: dorsal border more cranial in MRI than MER, lateral border more medial in MRI than MER.
Shamir, 2018[][13]	Retros	16 (7T)	Yes (CRW)	STN	Direct	High agreement (93%) between lead location on 7T images and MER.
Bot, 2019[3]	Retros	19 (7T)	Yes (Leksell)	STN	Mixed	The dorsal border of STN in 7T MRI was located more cranially than MER-based dorsal border.
Verlaat, 2024[20]	Retros	75 (1.5/3T group) 182 (7T group)	Yes (Leksell)	STN	Mixed	7T targeting enabled superior visual alignment of trajectories to the dorsolateral STN, resulting in longer MER and lower channels used. Similar clinical outcome.

GPi, globus pallidus interna; LEDD, levodopa equivalent daily dose; MER, microelectrode recording; NA, not available; Pros, prospective; Retros, retrospective; RN, red nucleus; STN, subthalamic nucleus

All studies suggested an improved visualization of target (mainly STN) using 3T MRI; two of them specifically reported an increased sharpness of target border [4, 14], with only one study semiquantitatively scoring the clearness of STN borders in order to demonstrate the superiority of 3T over 1.5T MRI [4]. One study assessed the image fusion accuracy, reporting no difference between 1.5T and 3T MRI [21]. Two studies compared the STN coordinates obtained with 1.5T vs. 3T MR-based targeting [8, 21], again finding no significant differences. Accuracy of targeting was evaluated in four studies, using different MER-based parameters, and with conflicting results. In detail, three studies, dealing with 92 patients overall (42 operated using 1.5T vs. 50 using 3T) reported an increase in targeting accuracy using 3T MRI [4, 17, 21]. Instead, a single study on 27 patients found no difference between the two groups [14]. This latter study was the only one adopting a frameless technique using intraoperative MRI for targeting. To note, phantom studies demonstrated that the use of the frame causes remarkable image distortion if scanned at 3T [12]; in frame-based procedures, indeed, MRI was performed before frame positioning. Surgical time was found to be reduced in 3T group in one study [4] and to be not different to 1.5T in another one [14]. Finally, and most importantly, all 3 studies assessing a clinical outcome endpoint agreed that no difference between 1.5T and 3T-based targeting can be demonstrated [4, 17, 21]. As for safety, no major concerns were reported [4, 17]. Intriguigly, Cheng et al. [4] suggested that the use of 3T MR could be associated with a reduced rate of infection, due to the increased accuracy leading to a reduced number of trajectories and thus to a shorter procedure. We found no data on the incidence of stimulation-induced adverse effects in patients operated using 1.5T or 3T-based targeting.

### Perspectives

One major finding of our systematic review is the lack of well-designed prospective studies aimed at solving the issue on the usefulness of 3T MRI in DBS targeting. Instead, in latest years, in parallel with the wider availability of high-field MRI, and starting from a pioneer phantom work showing acceptable image distortion [5], some papers have focused on the incorporation of 7T MRI in DBS workflow (Table 3). However, also in this case the amount of evidence is limited. A recent large comparative study demonstrated an improved precision of STN targeting using 7T MRI compared to 1.5T or 3T MRI [20], overturning older results on a much smaller case series [18]. However, clinical outcome was not affected by the MRI field strength [20], similarly to the findings of our systematic review. Interestingly, some Authors found discrepancies in the location of STN comparing anatomical image on 7T MRI and functional MER signal. In particular, the anatomical dorsal border of STN is apparently more rostrally located as compared to functional STN dorsal border [3, 19]. This finding, though not confirmed by all Authors [13], underscores the role of MER also in the era of very high-field MRI. In fact, beyond anatomical visualization, an effective STN DBS involves targeting of its motor area, actually corresponding to the dorsolateral electrophysiological STN with increased β-oscillatory activity [3].

### Limitations of the present study

As limitations of the study, as already mentioned, we acknowledge: *(i)* the low number of studies comparatively addressing 1.5T vs. 3T MR-based targeting; *(ii)* the quality of evidence, which is non-negligibly biased (Fig. 2).

## Conclusions

Innovation has a key role in DBS targeting. The use of high-field MRI, i.e. 3T and 7T, surely improves the ability of the surgeon to identify directly the deep grey nuclei, and therefore fosters surgical confidence. However, 1.5T MR imaging is adequate to perform a safe and effective DBS. In this scenario, physiological assessment using MER is of great help in identifying the functional areas of the anatomic nuclei, which are the actual best targets.

## Data Availability

No datasets were generated or analysed during the current study.

## Abbreviations

Gpi: Globus Pallidus Interna
LEDD: Levodopa Equivalent Daily Dose
MCP: Midcommissural Point
MER: Microelectrode Recording
MRI: Magnetic Resonance Imaging
PD: Parkinson’s Disease
PICO: Population, Intervention, Comparison, Outcomes
PRISMA: Preferred Reporting Items for Systematic Reviews and Meta-Analyses
Pros: Prospective
Retros: Retrospective
RN: Red Nucleus
STN: Subthalamic Nucleus
UPDRS: Unified Parkinson’s Disease Rating Scale
